# Supplementary material for: Theta-burst TMS of lateral occipital cortex reduces BOLD responses across category-selective areas in ventral temporal cortex
Source: Neuroimage. Author manuscript; Available in PMC 2021 May 4. (PMC8094793; doi:10.1016/j.neuroimage.2021.117790)
Supplement: 1 [file NIHMS1696743-supplement-1.pdf]

Supplementary Material

accompanying Groen, Silson, Pitcher, Baker (2021) “Theta-burst TMS of lateral occipital cortex reduces BOLD responses across category-selective areas in ventral temporal cortex”.

Table of contents

Supplementary Methods **2**

1. TMS-fMRI session counterbalancing scheme 2
2. Behavioral data analysis 2

Supplementary Results **3**

1. ROI analysis: full statistical tables for omnibus ANOVAs 3
    1.1 ANOVA results for scene-selective OPA and PPA 3
    1.2 ANOVA results for face-selective OFA and FFA 4
    1.3 ANOVA results for other ROIs: MPA and V1 5
2. ROI analysis: linear mixed effect models 6
    2.1 Linear mixed effects model comparison 7
        2.1.1 Random effects: R lmer formulas for model comparison 7
        2.1.2 Random effects: Likelihood ratio tests 8
        2.1.3 Fixed effects: R lmer formulas for model comparison 8
        2.1.4 Fixed effects: Likelihood ratio tests 9
    2.2 ANOVA results for model with random slopes 10
        2.2.1 OPA and PPA 10
        2.2.2 FFA and OFA 11
        2.2.3 MPA and V1 12
3. Whole brain analysis: additional linear mixed effects results 13
4. Behavioral data analysis: linear mixed effects results 14

Supplementary Figures **16**

S1: Target and localizer ROI accuracy 16
S2: ROI analysis for MPA and V1 17
S3: Whole brain linear mixed effects in the left hemisphere 18
S4: Whole brain main effects 19
S5: Whole brain linear mixed effects with random slopes 20
S6: Behavioral data (d’ and reaction times) 21
S7: Variability in stimulation sites across participants 22
S8: Proximity of OFA and the venous eclipse 23

Supplementary References **24**

Supplementary Methods

1. TMS-fMRI session counterbalancing scheme

	**stimulation**			
**subject #**	OPA	OFA	Sham	run order
**1**	1	2	3	2301
**2**	1	2	3	3012
**3**	1	2	3	0123
**4**	1	2	3	3021
**5**	2	1	3	1230
**6**	2	1	3	1032
**7**	2	1	3	2103
**8**	2	1	3	3210
**9**	3	2	1	0321
**10**	3	2	1	2301
**11**	3	2	1	3012
**12**	3	2	1	0123
**13**	2	3	1	1230
**14**	2	3	1	1032
**15**	2	3	1	2103
**16**	2	3	1	3210

2. Behavioral data analysis

The recorded behavioral responses were used to calculate discriminability measure d' (MacMillan & Creelman, 1991). In this analysis, correct responses to image repeats (occurring between 1-3 times for each stimulus block) were scored as Hits, while button presses to non-repeats were scored as False Alarms. Responses were marked as correct if the button press occurred after the onset of the repeated image before the onset of the third following image presentation (resulting in a 2400ms response window). Responses made outside that window but during stimulus presentation were marked as incorrect. Reaction times (RTs) were calculated based on the correct trials and reflected the time elapsed between the button press and the onset of the repeated image. Discriminability values and RTs were calculated separately for each subject, category and run. Scores of 1.0 or 0.0 for hits and false alarms for a given category within a run were subjected to a standard correction whereby half a trial was either subtracted or added to the actual score. The resulting d' and RT measures were fitted with a linear mixed effects model incorporating the same factors as the main fMRI analyses

(Fixed effects: TMS condition, Session, Run and Stimulus; random effects: subject). The behavioral data and R analysis script are provided on the OSF (https://osf.io/6nq7r/).

Supplementary Results

1. ROI analysis: full statistical tables for omnibus ANOVAs

The tables show the statistical output for the omnibus ANOVAs that were run on the fitted linear mixed effects models to identify potential effects of TBS in OPA and PPA (**section 1.1**; associated data in **Figure 2**), OFA and FFA (**section 1.2**; **Figure 3**) and MPA and V1 (**section 1.3**; **Figure S1**).

1.1 ANOVA results for scene-selective OPA and PPA

Type III Analysis of Variance Table with Satterthwaite's method							
	Sum Sq	Mean Sq	NumDF	DenDF	F value	Pr(>F)	Pr(>F) bonf
OPA							
TMScondition	0.00	0.00	2	2857	0.02	0.979	
Session	0.01	0.01	1	2857	0.07	0.796	
**Stimulus**	**246.06**	**35.15**	**7**	**2857**	**370.99**	**0.000**	**0.000**
**Run**	**21.76**	**7.25**	**3**	**2857**	**76.56**	**0.000**	**0.000**
TMScondition:Session	0.18	0.09	2	2857	0.93	0.395	
TMScondition:Stimulus	1.00	0.07	14	2857	0.75	0.719	
Session:Stimulus	0.48	0.07	7	2857	0.72	0.657	
TMScondition:Run	0.93	0.16	6	2857	1.64	0.133	
Session:Run	0.98	0.33	3	2857	3.44	0.016	0.097
Stimulus:Run	1.14	0.05	21	2857	0.57	0.938	
TMScondition:Session: Stimulus	0.81	0.06	14	2857	0.61	0.860	
TMScondition:Session:Run	0.49	0.08	6	2857	0.87	0.518	
TMSconditionStimulus:Run	1.50	0.04	42	2857	0.38	1.000	
Session:Stimulus:Run	1.40	0.07	21	2857	0.70	0.835	
TMScondition:Session: Stimulus:Run	1.29	0.03	42	2857	0.33	1.000	
PPA							
**TMScondition**	**0.85**	**0.42**	**2**	**2857**	**5.48**	**0.004**	**0.025**
**Session**	**2.46**	**2.46**	**1**	**2857**	**31.93**	**0.000**	**0.000**
**Stimulus**	**266.31**	**38.04**	**7**	**2857**	**493.93**	**0.000**	**0.000**
**Run**	**26.63**	**8.88**	**3**	**2857**	**115.25**	**0.000**	**0.000**
**TMScondition:Session**	**1.41**	**0.70**	**2**	**2857**	**9.14**	**0.000**	**0.001**

TMScondition:Stimulus	1.19	0.09	14	2857	1.10	0.348	
Session:Stimulus	0.12	0.02	7	2857	0.23	0.979	
TMScondition:Run	0.60	0.10	6	2857	1.30	0.255	
Session:Run	0.96	0.32	3	2857	4.14	0.006	0.037
Stimulus:Run	1.79	0.09	21	2857	1.11	0.333	
TMScondition:Session: Stimulus	0.28	0.02	14	2857	0.26	0.997	
TMScondition:Session:Run	0.43	0.07	6	2857	0.94	0.467	
TMScondition: Stimulus:Run	1.44	0.03	42	2857	0.44	0.999	
Session:Stimulus:Run	1.05	0.05	21	2857	0.65	0.884	
TMScondition:Session: Stimulus:Run	1.38	0.03	42	2857	0.43	1.000	

1.2 ANOVA results for face-selective OFA and FFA

Type III Analysis of Variance Table with Satterthwaite's method							
	Sum Sq	Mean Sq	NumDF	DenDF	F value	Pr(>F)	Pr(>F) bonf
OFA							
**TMScondition**	**1.33**	**0.67**	**2**	**2857**	**5.92**	**0.003**	**0.016**
Session	0.45	0.45	1	2857	4.01	0.045	0.272
**Stimulus**	**121.17**	**17.31**	**7**	**2857**	**153.77**	**0.000**	**0.000**
**Run**	**32.79**	**10.93**	**3**	**2857**	**97.09**	**0.000**	**0.000**
TMScondition:Session	0.12	0.06	2	2857	0.51	0.598	
TMScondition:Stimulus	1.58	0.11	14	2857	1.00	0.445	
Session:Stimulus	0.36	0.05	7	2857	0.46	0.865	
TMScondition:Run	0.59	0.10	6	2857	0.88	0.509	
**Session:Run**	**2.07**	**0.69**	**3**	**2857**	**6.14**	**0.000**	**0.002**
Stimulus:Run	2.43	0.12	21	2857	1.03	0.426	
TMScondition:Session: Stimulus	0.68	0.05	14	2857	0.43	0.964	
TMScondition:Session:Run	0.87	0.15	6	2857	1.29	0.258	
TMScondition:Stimulus:Run	2.52	0.06	42	2857	0.53	0.994	
Session:Stimulus:Run	1.56	0.07	21	2857	0.66	0.875	
TMScondition:Session: Stimulus:Run	2.08	0.05	42	2857	0.44	0.999	
FFA							
**TMScondition**	**2.60**	**1.30**	**2**	**2857**	**7.84**	**0.000**	**0.002**

Session	0.69	0.69	1	2857	4.18	0.041	0.246
**Stimulus**	**192.97**	**27.57**	**7**	**2857**	**166.46**	**0.000**	**0.000**
**Run**	**32.87**	**10.96**	**3**	**2857**	**66.16**	**0.000**	**0.000**
**TMScondition:Session**	**3.75**	**1.88**	**2**	**2857**	**11.33**	**0.000**	**0.000**
TMScondition:Stimulus	2.33	0.17	14	2857	1.00	0.445	
Session:Stimulus	0.79	0.11	7	2857	0.68	0.692	
TMScondition:Run	0.72	0.12	6	2857	0.73	0.627	
**Session:Run**	**2.99**	**1.00**	**3**	**2857**	**6.02**	**0.000**	**0.003**
Stimulus:Run	3.03	0.14	21	2857	0.87	0.629	
TMScondition:Session: Stimulus	0.58	0.04	14	2857	0.25	0.998	
TMScondition:Session:Run	0.41	0.07	6	2857	0.41	0.872	
TMScondition:Stimulus:Run	3.26	0.08	42	2857	0.47	0.999	
Session:Stimulus:Run	2.74	0.13	21	2857	0.79	0.739	
TMScondition:Session: Stimulus:Run	2.44	0.06	42	2857	0.35	1.000	

1.3 ANOVA results for other ROIs: MPA and V1

Type III Analysis of Variance Table with Satterthwaite's method							
	Sum Sq	Mean Sq	NumDF	DenDF	F value	Pr(>F)	Pr(>F) bonf
MPA							
TMScondition	0.27	0.14	2	2857	1.46	0.233	
**Session**	**2.44**	**2.43**	**1**	**2857**	**26.23**	**0.000**	**0.000**
**Stimulus**	**186.88**	**26.70**	**7**	**2857**	**287.64**	**0.000**	**0.000**
**Run**	**11.80**	**3.93**	**3**	**2857**	**42.37**	**0.000**	**0.000**
TMScondition:Session	0.20	0.10	2	2857	1.07	0.345	
TMScondition:Stimulus	1.60	0.11	14	2857	1.23	0.247	
Session:Stimulus	0.27	0.04	7	2857	0.41	0.895	
TMScondition:Run	0.97	0.16	6	2857	1.74	0.109	
Session:Run	0.22	0.07	3	2857	0.78	0.507	
Stimulus:Run	2.09	0.10	21	2857	1.07	0.372	
TMScondition:Session: Stimulus	0.90	0.06	14	2857	0.69	0.785	
TMScondition:Session:Run	0.88	0.15	6	2857	1.58	0.150	
TMScondition:Stimulus:Run	2.00	0.05	42	2857	0.51	0.996	
Session:Stimulus:Run	1.54	0.07	21	2857	0.79	0.734	
TMScondition:Session: Stimulus:Run	2.64	0.06	42	2857	0.68	0.944	

V1							
**TMScondition**	**1.32**	**0.66**	**2**	**2857**	**4.88**	**0.008**	**0.046**
Session	0.00	0.00	1	2857	0.01	0.913	
**Stimulus**	**32.62**	**4.66**	**7**	**2857**	**34.37**	**0.000**	**0.000**
**Run**	**16.74**	**5.58**	**3**	**2857**	**41.15**	**0.000**	**0.000**
TMScondition:Session	0.36	0.18	2	2857	1.32	0.267	
TMScondition:Stimulus	0.91	0.07	14	2857	0.48	0.945	
Session:Stimulus	0.27	0.04	7	2857	0.28	0.962	
TMScondition:Run	0.97	0.16	6	2857	1.20	0.305	
Session:Run	1.59	0.53	3	2857	3.91	0.008	0.051
Stimulus:Run	2.87	0.14	21	2857	1.01	0.447	
TMScondition:Session:Stimulus	1.21	0.09	14	2857	0.64	0.835	
TMScondition:Session:Run	0.46	0.08	6	2857	0.56	0.761	
TMScondition:Stimulus:Run	2.75	0.07	42	2857	0.48	0.998	
Session:Stimulus:Run	2.11	0.10	21	2857	0.74	0.793	
TMScondition:Session:Stimulus:Run	2.19	0.05	42	2857	0.39	1.000	

2. ROI analysis: linear mixed effect models

Our linear mixed effects model specification included a random intercept for participants, allowing for the statistical model to take into account the high inter-participant variability in overall fMRI response. While less often applied in neuroimaging research, mixed effects models can incorporate more complex random effects structure, including random slopes for each within-subject factor and their interactions, allowing the model to take into account variability across participants from session to session, for example, or variability in participant's sensitivity to the experimental manipulation. We explored the inclusion of these factors in our ROI analyses using a model selection procedure (Meteyard & Davies, 2020; Brown 2020), whereby increasingly complex random effects structures were fitted first followed by the inclusion of the fixed effects, and model selection was evaluated using likelihood ratio tests (LRTs; see section 2.1. The results suggested that the data would be better fit (higher likelihood) by a model that additionally included random slopes for Session and TMS-condition, but not their interaction, for a all of the ROIs (see **section 2.1** below for a full report of the model selection and associated tests, and OSF page https://osf.io/6nq7r for the R script used for the model comparison).

Importantly, however, the overall pattern of results was qualitatively identical to that reported in the simpler, random intercept-only model, indicating TBS-induced response reductions in PPA and FFA but not OPA, OFA, MPA or V1 (see **section 2.2** below). A whole brain 3dLME analysis with the random effects structure that best fitted the ROI data also showed a qualitatively similar pattern to the main analysis (see **section 3** and **Figure S5**).

2.1 Linear mixed effects model comparison

Following recommendations outlined in Meteyard & Davies (2020), we started with fitting random effects only (no fixed effects) to identify the best-fitting random effects structure. The respective model specifications are presented in **section 2.1.1**. The first model includes only a random intercept for subject; the second and third models add random slopes for TMScondition (OPA, OFA, Sham) and Session (Pre vs. Post), respectively. Additional models that included the interaction for Session and TMScondition or additional random effects resulted in singular or non converging models for all of the ROIs, and are therefore marked in gray. LRT results for each ROI comparing models 1,2 and 3 are presented in **section 2.1.2**. At the next step, we added fixed effects while keeping the random effects structure constant. The reference model (Model 1) contained no fixed effects, while model 2 included the manipulated within-subject factors. Finally, model 3 additionally included a fixed effect of Run. Model 3 performed resulted in the best fits in all ROIs. The model specifications and LRT results for each ROI are presented in **sections 2.1.3** and **2.1.4**, respectively.

2.1.1 Random effects: R lmer formulas for model comparison

**Model name**	**Description**	**Formula**
Model R0	random intercept only	lmer(fMRIresponse ~ 1 + (1|subject)
Model R1	random intercept and slope for TMScondition	lmer(fMRIresponse ~ 1 + (TMScondition|subject)
Model R2	random intercept and slope for TMScondition and Session	lmer(fMRIresponse ~ 1 + (TMScondition+Session|subject)
Model R3	random intercept and slope for TMS condition and Session interaction	lmer(fMRIresponse ~ 1 + (TMScondition*Session|subject)
Model R4	random intercept and slope for TMScondition and Session and Run	lmer(fMRIresponse ~ 1 + (TMScondition+Session+Run|subject)
Model R5	random intercept and slope for TMScondition and Session and Stimulus	lmer(fMRIresponse ~ 1 + (TMScondition+Session+Stimulus|subject)
Model R6	random intercept and slopes for all within-subject factors	lmer(fMRIresponse ~ 1 + (TMScondition+Session+Run+Stimulus|subject)

2.1.2 Random effects: Likelihood ratio tests

	Df	AIC	BIC	logLik	deviance	Chisq	Df	Pr(>Chisq)
OPA								
Model R0	3	3514.2	3532.3	-1754.1	3508.2			
Model R1	8	3456.7	3504.9	-1720.3	3440.7	67.5	5	**3.4E-13**
Model R2	12	3444.6	3516.9	-1710.3	3420.6	20.1	4	**4.8E-04**
PPA								
Model R0	3	3401.4	3419.5	-1697.7	3395.4			
Model R1	8	3331	3379.2	-1657.5	3315.0	80.4	5	**7.0E-16**
Model R2	12	3316.8	3389.1	-1646.4	3292.8	22.2	4	**1.8E-04**
OFA								
Model R0	3	3187.9	3206	-1591	3181.9			
Model R1	8	3111	3159.2	-1547.5	3095.0	86.9	5	**2.2E-16**
Model R2	12	3093.2	3165.5	-1534.6	3069.2	25.8	4	**3.5E-05**
FFA								
Model R0	3	4385.4	4403.5	-2189.7	4379.4			
Model R1	8	4124.2	4172.4	-2054.1	4108.2	271.2	5	**2.20E-16**
Model R2	12	4105.9	4178.2	-2041	4081.9	26.3	4	**2.75E-05**
MPA								
Model R0	3	3102.9	3121	-1548.5	3096.9			
Model R1	8	3080.1	3128.3	-1532.1	3064.1	32.778	5	**4.17E-06**
Model R2	12	3071.9	3144.2	-1523.9	3047.9	16.26	4	**0.002689**
V1								
Model R0	3	2965.2	2983.3	-1479.6	2959.2			
Model R1	8	2770.6	2818.8	-1377.3	2754.6	204.6	5	**2.20E-16**
Model R2	12	2685.3	2757.7	-1330.7	2661.3	93.237	4	**2.20E-16**

2.1.3 Fixed effects: R lmer formulas for model comparison

**Model name**	**Description**	**Formula**
Model F0	no fixed effects w/ best fitting random effects (model R2;see 2.1.2) *	lmer(fMRIresponse ~ 1 + (TMScondition+Session|subject)

Model F1	fixed effects for experimentally manipulated within-subject factors	lmer(fMRIresponse ~ 1 + Stimulus*Session*TMScondition + (TMScondition+Session|subject)
Model F2	fixed effects for experimentally manipulated within-subject factors + Run	lmer(fMRIresponse ~ 1 + Stimulus*Session*TMScondition*Run + (TMScondition+Session|subject)

* Models R2 and F0 have identical model formulas, but their estimated coefficients are not numerically identical, because the models to compare fixed effects were fitted with maximum likelihood estimation (ML) rather than restricted maximum likelihood (REML) (see Brown, 2020; Meteyard and Davies, 2020).

2.1.4 Fixed effects: Likelihood ratio tests

	Df	AIC	BIC	logLik	deviance	Chisq	Df	Pr(>Chisq)
OPA								
Model F0	12	3440.5	3512.9	-1708.3	3416.5			
Model F1	59	1550.5	1906.1	-716.3	1432.5	1984	47	**2.20E-16**
Model F2	203	1481	2704.6	-537.5	1075	357.52	144	**2.20E-16**
PPA								
Model F0	12	3313.3	3385.6	-1644.6	3289.3			
Model F1	59	1017.3	1372.9	-449.6	899.3	2390	47	**2.20E-16**
Model F2	203	796.57	2020.2	-195.3	390.57	508.69	144	**2.20E-16**
OFA								
Model F0	12	3089.2	3161.6	-1532.6	3065.2			
Model F1	59	2229.3	2584.9	-1055.7	2111.3	953.95	47	**2.20E-16**
Model F2	203	2083.5	3307.1	-838.8	1677.5	433.76	144	**2.20E-16**
FFA								
Model F0	12	4103.2	4175.6	-2039.6	4079.2			
Model F1	59	3048.3	3403.9	-1465.2	2930.3	1148.9	47	**2.20E-16**
Model F2	203	2976.4	4200.0	-1285.2	2570.4	359.86	144	**2.20E-16**
MPA								
Model F0	12	3067.6	3139.9	-1521.8	3043.6			
Model F1	59	1543.6	1899.2	-712.8	1425.6	1618	47	**2.20E-16**
Model F2	203	1571.4	2795.0	-582.7	1165.4	260.19	144	**1.04E-08**
V1								
Model F0	12	2683.3	2755.7	-1329.7	2659.3			
Model F1	59	2492.9	2848.5	-1187.4	2374.9	284.46	47	**2.20E-16**
Model F2	203	2519.8	3743.4	-1056.9	2113.8	261.07	144	**8.49E-09**

2.2 ANOVA results for model with random slopes

This section contains the full omnibus ANOVA statistical results for the best-fitting alternative linear mixed effects model that includes all four fixed effects plus random slopes for TMScondition and Session for ROIs OPA and PPA **(2.2.1)**, FFA and OFA (**2.2.2**) and MPA and V1 **(2.2.3)**.

2.2.1 OPA and PPA

Type III Analysis of Variance Table with Satterthwaite's method							
	Sum Sq	Mean Sq	NumDF	DenDF	F value	Pr(>F)	Pr(>F) bonf
OPA							
TMScondition	0.00	0.00	2	15	0.01	0.992	
Session	0.00	0.00	1	15	0.00	0.957	
**Stimulus**	**246.06**	**35.15**	**7**	**2812**	**420.00**	**0.000**	**0.000**
**Run**	**22.08**	**7.36**	**3**	**2812**	**87.95**	**0.000**	**0.000**
TMScondition:Session	0.17	0.08	2	2812	0.98	0.374	
TMScondition:Stimulus	1.00	0.07	14	2812	0.85	0.609	
Session:Stimulus	0.48	0.07	7	2812	0.81	0.577	
TMScondition:Run	0.95	0.16	6	2812	1.90	0.078	.
Session:Run	0.89	0.30	3	2812	3.54	0.014	0.084
Stimulus:Run	1.14	0.05	21	2812	0.65	0.884	
TMScondition:Session:Stimulus	0.81	0.06	14	2812	0.69	0.786	
TMScondition:Session:Run	0.50	0.08	6	2812	1.00	0.424	
TMScondition:Stimulus:Run	1.50	0.04	42	2812	0.43	1.000	
Session:Stimulus:Run	1.40	0.07	21	2812	0.80	0.729	
TMScondition:Session:Stimulus:Run	1.29	0.03	42	2812	0.37	1.000	
PPA							
TMScondition	0.05	0.03	2	15	0.40	0.680	
Session	0.39	0.39	1	15	5.88	0.028	0.171
**Stimulus**	**266.31**	**38.04**	**7**	**2812**	**571.44**	**0.000**	**0.000**
**Run**	**27.01**	**9.00**	**3**	**2812**	**135.21**	**0.000**	**0.000**
**TMScondition:Session**	**1.32**	**0.66**	**2**	**2812**	**9.93**	**0.000**	**0.000**
TMScondition:Stimulus	1.19	0.09	14	2812	1.28	0.213	
Session:Stimulus	0.12	0.02	7	2812	0.26	0.969	
TMScondition:Run	0.54	0.09	6	2812	1.36	0.228	
**Session:Run**	**0.90**	**0.30**	**3**	**2812**	**4.50**	**0.004**	**0.022**
Stimulus:Run	1.79	0.09	21	2812	1.28	0.176	

TMScondition:Session:Stimulus	0.28	0.02	14	2812	0.30	0.994	
TMScondition:Session:Run	0.43	0.07	6	2812	1.09	0.368	
TMScondition:Stimulus:Run	1.44	0.03	42	2812	0.51	0.996	
Session:Stimulus:Run	1.05	0.05	21	2812	0.75	0.781	
TMScondition:Session:Stimulus:Run	1.38	0.03	42	2812	0.49	0.997	

2.2.2 FFA and OFA

Type III Analysis of Variance Table with Satterthwaite's method							
	Sum Sq	Mean Sq	NumDF	DenDF	F value	Pr(>F)	Pr(>F) bonf
OFA							
**TMScondition**	**0.19**	**0.09**	**2**	**15**	**0.91**	**0.425**	
Session	0.09	0.09	1	15	0.85	0.372	
**Stimulus**	**121.17**	**17.31**	**7**	**2812**	**168.64**	**0.000**	**0.000**
**Run**	**32.78**	**10.93**	**3**	**2813**	**106.45**	**0.000**	**0.000**
TMScondition:Session	0.12	0.06	2	2813	0.56	0.570	
TMScondition:Stimulus	1.58	0.11	14	2812	1.10	0.350	
Session:Stimulus	0.36	0.05	7	2812	0.50	0.833	
TMScondition:Run	0.59	0.10	6	2813	0.97	0.447	
**Session:Run**	**2.07**	**0.69**	**3**	**2813**	**6.73**	**0.000**	**0.001**
Stimulus:Run	2.43	0.12	21	2812	1.13	0.312	
TMScondition:Session:Stimulus	0.68	0.05	14	2812	0.48	0.947	
TMScondition:Session:Run	0.87	0.15	6	2813	1.41	0.205	
TMScondition:Stimulus:Run	2.52	0.06	42	2812	0.59	0.985	
Session:Stimulus:Run	1.56	0.07	21	2812	0.72	0.812	
TMScondition:Session:Stimulus:Run	2.08	0.05	42	2812	0.48	0.998	
FFA							
TMScondition	0.41	0.20	2	15	1.50	0.255	
Session	0.13	0.13	1	15	0.93	0.350	
**Stimulus**	**192.97**	**27.57**	**7**	**2812**	**202.82**	**0.000**	**0.000**
**Run**	**33.09**	**11.03**	**3**	**2812**	**81.16**	**0.000**	**0.000**
**TMScondition:Session**	**3.64**	**1.82**	**2**	**2812**	**13.40**	**0.000**	**0.000**
TMScondition:Stimulus	2.33	0.17	14	2812	1.22	0.250	
Session:Stimulus	0.79	0.11	7	2812	0.82	0.567	

TMScondition:Run	0.74	0.12	6	2812	0.91	0.488	
**Session:Run**	**2.94**	**0.98**	**3**	**2812**	**7.21**	**0.000**	**0.000**
Stimulus:Run	3.03	0.14	21	2812	1.06	0.382	
TMScondition:Session:Stimulus	0.58	0.04	14	2812	0.31	0.993	
TMScondition:Session:Run	0.42	0.07	6	2812	0.52	0.796	
TMScondition:Stimulus:Run	3.26	0.08	42	2812	0.57	0.988	
Session:Stimulus:Run	2.74	0.13	21	2812	0.96	0.513	
TMScondition:Session:Stimulus :Run	2.44	0.06	42	2812	0.43	1.000	

2.2.3 MPA and V1

Type III Analysis of Variance Table with Satterthwaite's method							
	Sum Sq	Mean Sq	NumDF	DenDF	F value	Pr(>F)	Pr(>F) bonf
MPA							
TMScondition	0.06	0.03	2	15	0.35	0.713	
Session	0.67	0.67	1	15	7.70	0.014	0.085
**Stimulus**	**186.88**	**26.70**	**7**	**2812**	**306.47**	**0.000**	**0.000**
**Run**	**11.87**	**3.96**	**3**	**2812**	**45.43**	**0.000**	**0.000**
TMScondition:Session	0.19	0.10	2	2812	1.09	0.335	
TMScondition:Stimulus	1.60	0.11	14	2812	1.31	0.194	
Session:Stimulus	0.27	0.04	7	2812	0.44	0.877	
TMScondition:Run	0.99	0.16	6	2812	1.89	0.079	.
Session:Run	0.21	0.07	3	2812	0.80	0.493	
Stimulus:Run	2.09	0.10	21	2812	1.14	0.295	
TMScondition:Session:Stimulus	0.90	0.06	14	2812	0.74	0.738	
TMScondition:Session:Run	0.90	0.15	6	2812	1.72	0.111	
TMScondition:Stimulus:Run	2.00	0.05	42	2812	0.55	0.992	
Session:Stimulus:Run	1.54	0.07	21	2812	0.84	0.6672	
TMScondition:Session:Stimulus :Run	2.64	0.06	42	2812	0.72	0.9092	
V1							
TMScondition	0.08	0.04	2	15	0.36	0.703	
Session	0.00	0.00	1	15	0.00	0.971	
**Stimulus**	**32.62**	**4.66**	**7**	**2812**	**40.06**	**0.000**	**0.000**
**Run**	**16.74**	**5.58**	**3**	**2812**	**47.96**	**0.000**	**0.000**

TMScondition:Session	0.36	0.18	2	2812	1.53	0.216	
TMScondition:Stimulus	0.91	0.07	14	2812	0.56	0.897	
Session:Stimulus	0.27	0.04	7	2812	0.33	0.942	
TMScondition:Run	0.97	0.16	6	2812	1.39	0.214	
Session:Run	1.59	0.53	3	2812	4.55	0.003	**0.021**
Stimulus:Run	2.87	0.14	21	2812	1.18	0.261	
TMScondition:Session:Stimulus	1.21	0.09	14	2812	0.74	0.731	
TMScondition:Session:Run	0.46	0.08	6	2812	0.65	0.687	
TMScondition:Stimulus:Run	2.75	0.07	42	2812	0.56	0.990	
Session:Stimulus:Run	2.11	0.10	21	2812	0.86	0.640	
TMScondition:Session:Stimulus:Run	2.19	0.05	42	2812	0.45	0.999	

3. Whole brain analysis: additional linear mixed effects results

Although our initial whole brain linear mixed effects analyses (implemented in 3dLME) included participant as a random factor, we also ran an additional model that included both Session and TMS condition as random factors. These additional factors were included based on the model comparisons performed at the ROI level (see **section 2** above).

The additional modelling of Session and TMS condition as random factors had little impact on the pattern of results at the whole brain level. Indeed, the spatial distribution of the Session by TMS condition interaction closely resembles the original 3dLME output (see **Figure S5A**). Specifically, significant Session by TMS condition interactions were present locally within rOPA, as well as in close proximity to rOFA. The downstream effects were also largely similar, with a prominent interaction effect present in anterior portions of PPA and to a lesser extent in posterior PPA. The previous cluster overlapping FFA however was not present (at the same statistical threshold).

For consistency, we also examined the direction of the effect of Session (Pre, Post) for each TBS condition separately (**Figure S5B-D)**. Again, results were largely consistent with our previous analyses. For example, TBS of rOPA elicited a local reduction within rOPA, rOFA and anterior PPA, whereas TBS of rOFA resulted in positive responses in both rOFA and rOPA, but an even more pronounced reduction in anterior PPA. During sham, the vast majority of responses were positive, consistent with our prior analyses.

4. Behavioral data analysis: linear mixed effects results

Type III Analysis of Variance Table with Satterthwaite's method						
	Sum Sq	Mean Sq	NumDF	DenDF	F value	Pr(>F)
d'						
TMScondition	6.42	3.209	2	2706.4	1.6908	0.1846
Session	1.81	1.808	1	2703.9	0.9522	0.3292
**stimCondition**	**442.87**	**63.266**	**7**	**2703.9**	**33.3299**	**2.20E-16**
**Run**	**114.47**	**38.157**	**3**	**2704**	**20.1017**	**6.97E-13**
TMScondition:Session	4.83	2.414	2	2703.9	1.2716	0.2806
TMScondition:stimCondition	31.82	2.273	14	2703.9	1.1974	0.2698
Session:stimCondition	14.24	2.035	7	2703.9	1.0719	0.3787
TMScondition:Run	6.86	1.144	6	2703.9	0.6027	0.7285
Session:Run	4.52	1.507	3	2703.9	0.794	0.4971
stimCondition:Run	34.34	1.635	21	2703.9	0.8615	0.643
TMScondition:Session:stimCondition	13.06	0.933	14	2703.9	0.4914	0.939
TMScondition:Session:Run	8.99	1.498	6	2703.9	0.7893	0.5782
TMScondition:stimCondition:Run	56.58	1.347	42	2703.9	0.7097	0.9199
Session:stimCondition:Run	29.61	1.41	21	2703.9	0.7427	0.7913
TMScondition:Session:stimCondition:Run	50.71	1.207	42	2703.9	0.636	0.9674
RT						
TMScondition	47611	23805	2	2505.3	1.0792	0.34003
**Session**	**453174**	**453174**	**1**	**2502.2**	**20.5439**	**6.10E-06**
stimCondition	280777	40111	7	2502	1.8184	0.07952
Run	28387	9462	3	2502.1	0.429	0.73227
TMScondition:Session	112869	56434	2	2502	2.5584	0.07763
TMScondition:stimCondition	431338	30810	14	2502	1.3967	0.14585
Session:stimCondition	138626	19804	7	2502	0.8978	0.50714
TMScondition:Run	27634	4606	6	2502.1	0.2088	0.97416
Session:Run	52183	17394	3	2502	0.7885	0.50019
stimCondition:Run	502210	23915	21	2502	1.0841	0.35746
TMScondition:Session:stimCondition	184268	13162	14	2502	0.5967	0.86976

TMScondition:Session:Run	63637	10606	6	2502	0.4808	0.82307
TMScondition:stimCondition:Run	792755	18875	42	2502	0.8557	0.73203
Session:stimCondition:Run	497599	23695	21	2502	1.0742	0.36888
TMScondition:Session:stimCondition:Run	999345	23794	42	2502	1.0787	0.33766

Supplementary Figures

S1: Target and localizer ROI accuracy

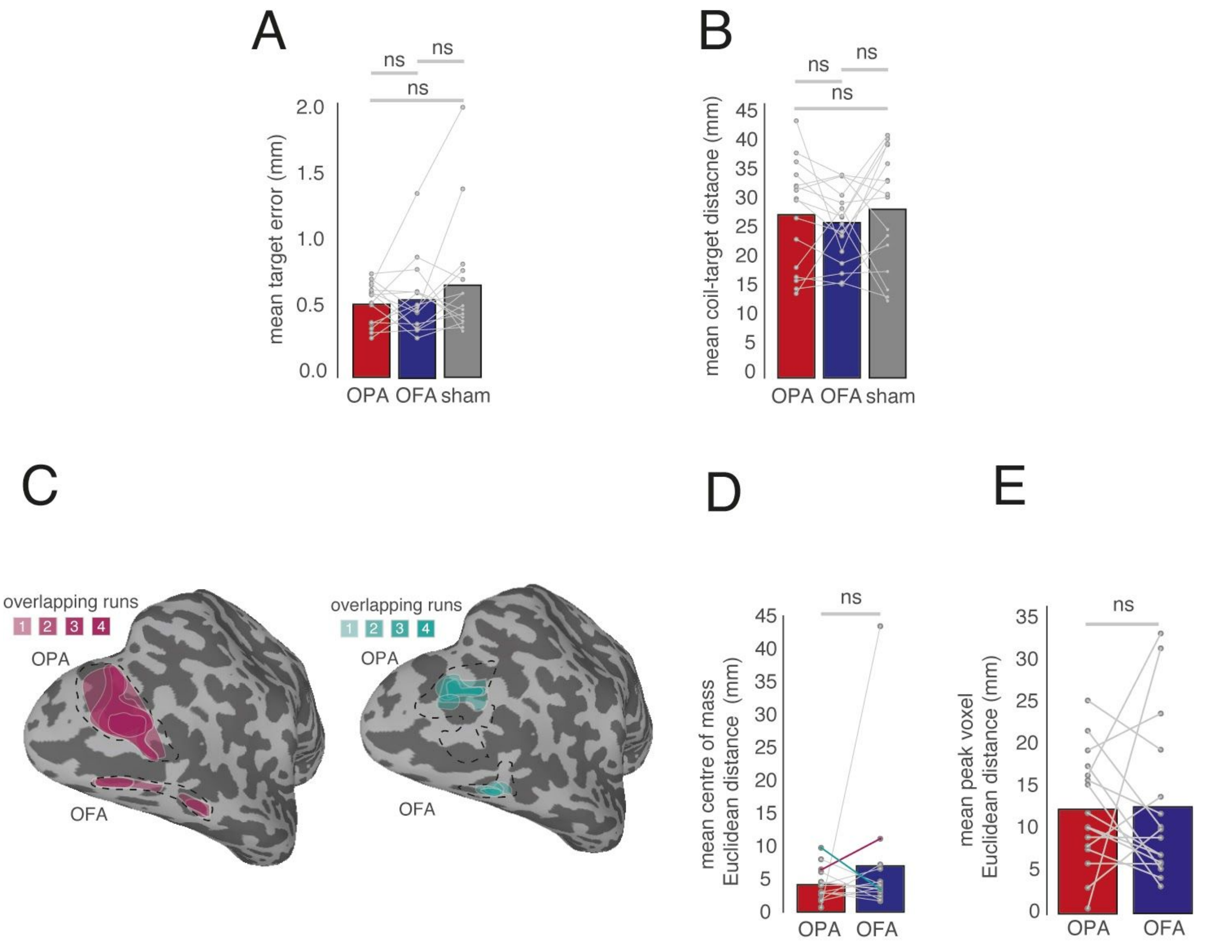

Figure S1: *Reliability of ROI localisation and TMS precision.* ***A,*** *Bars represent the mean coil-target error in each individual participant across the three TMS conditions. No pairwise comparisons were significant (p>0.05, in all cases).* ***B,*** *Bars represent the mean coil-target distance in each individual participant across the three TMS conditions. No pairwise comparisons were significant (p>0.05, in all cases).* ***C,*** *ROI localisation for two representative participants. In each case, the original ROIs (OPA, OFA) taken from the independent localiser session are shown by the black dashed line. For each participant, ROIs were defined for each pre TMS run separately and the areal extent of this run overlap is depicted by the saturation (1 run=light, 4 runs=dark). It is noteworthy that in each case the individual run ROIs all fall within the boundary of the original ROIs.* ***D,*** *Bars represent the mean Euclidean distance in the centre of mass between the original ROI and the average of the four pre TMS runs in each participant separately. On average, these distances were similar, despite one clear outlier. A paired t-test revealed no significant difference between ROIs (p>0.05).* ***E,*** *Bars represent the mean Euclidean distance between the target voxel and the peak voxel of selectivity in each ROI taken from the average of the pre TMS runs in each participant separately. Again, despite some variability these distances were largely equivalent across ROIs. A paired t-test revealed no significant difference between ROIs (p>0.05).*

S2: ROI analysis for MPA and V1

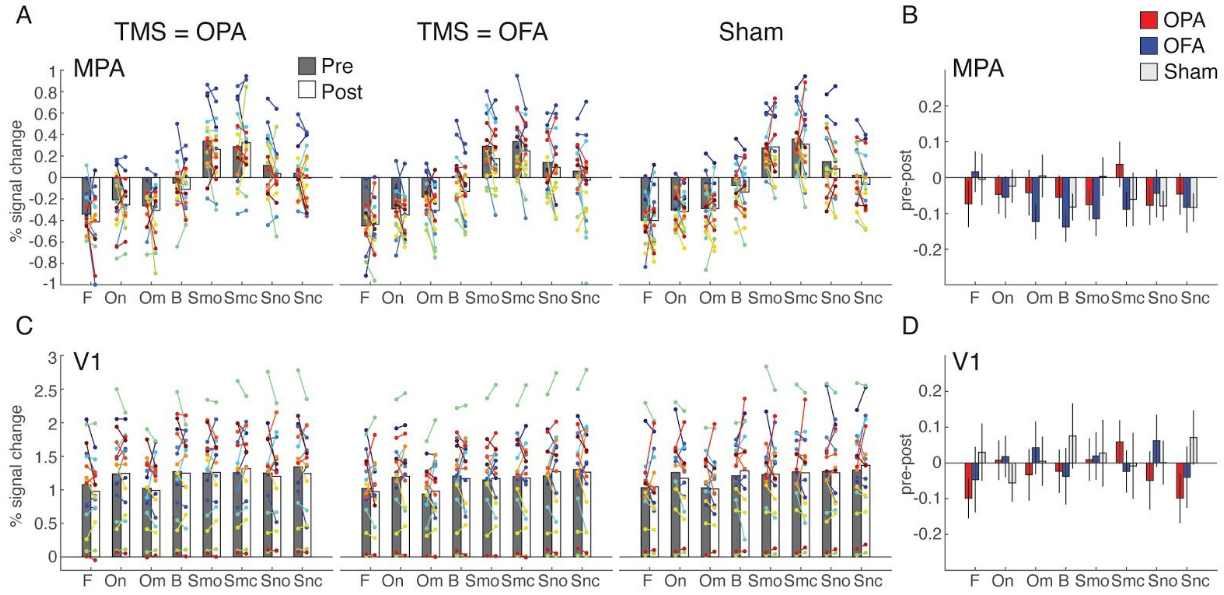

Figure S2: *TMS effects in MPA and V1.* ***A,*** *Bars represent the group-average response in MPA for each stimulus category during Pre (gray) and Post (white) sessions during TBS to OPA (left), TBS to OFA (middle) and the Sham (right). Individual participant data points are overlaid and linked in each case.* ***B,*** *Bars represent the response difference between the Pre and Post sessions for TMS to OPA (red), TMS to OFA (blue) and Sham (gray) in MPA. Error bars represent S.E.M. across subjects.* ***C-D,*** *same as A-B, but for V1. Note that in V1, two subjects have virtually no response to any stimulus category. Visual inspection showed that this is due to somewhat suboptimal alignment between the localizer and the TMS/fMRI sessions resulting in a small cutoff of activity in the tip of the occipital lobe which included the V1 ROI for these subjects. Statistical results were qualitatively identical when excluding these subjects from analysis.*

S3: Whole brain linear mixed effects in the left hemisphere

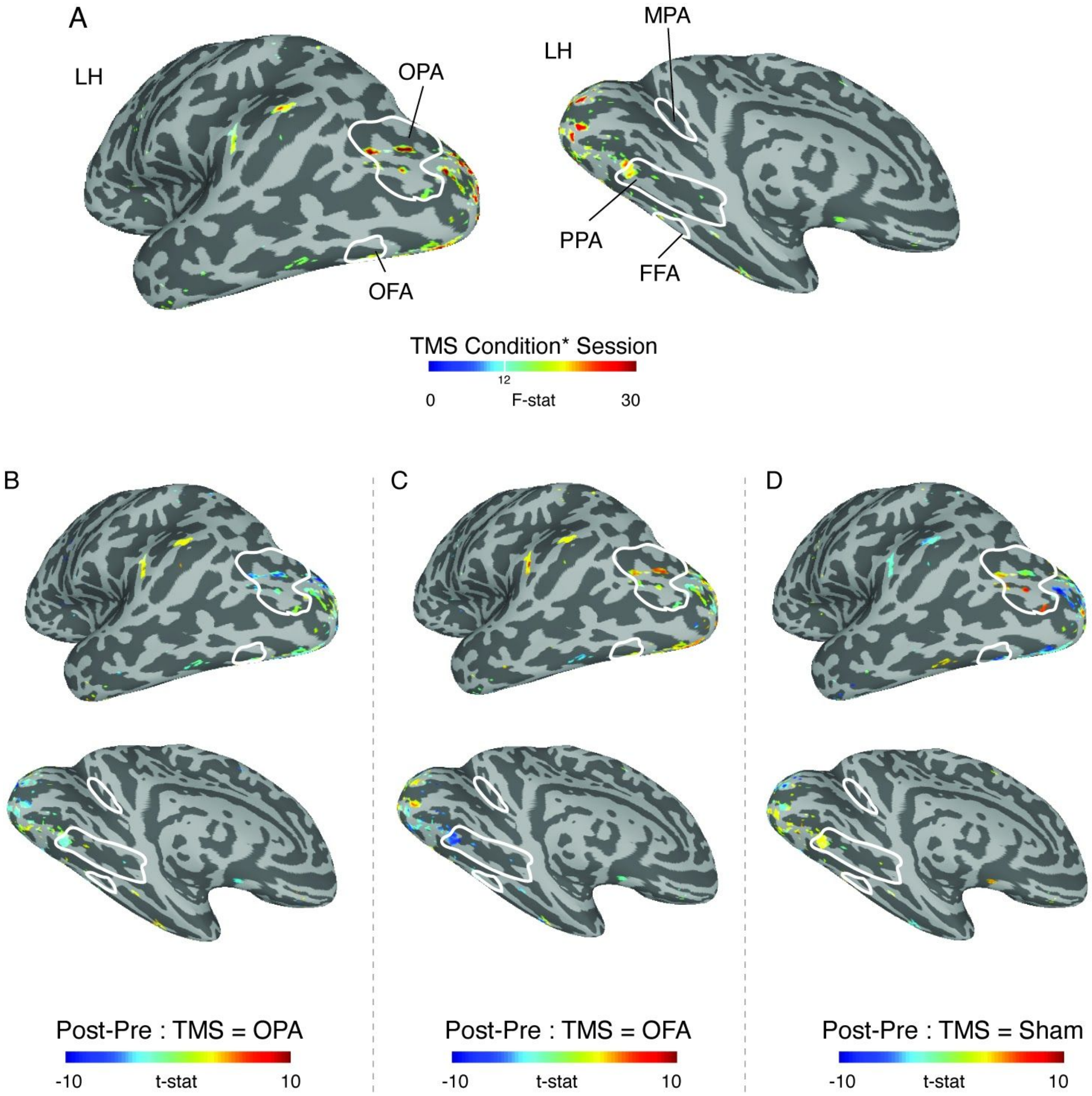

Figure S3: *Linear mixed effects in the left hemisphere.* ***A,*** *Locations of significant Session by TMS Condition interaction effects (p=2.4-6, q=4.2-5) are overlaid onto lateral (top) and medial (bottom) views of a partially inflated surface reconstruction of the left hemisphere (sulci=dark gray, gyri=light gray). Group-based definitions of OPA, OFA (top), PPA, MPA and FFA (bottom) are overlaid in white. Unlike the TMS'd hemisphere, significant interaction effects were both smaller in areal extent and lower in magnitude. Despite this, significant interaction effects were present within EVC, OPA and posterior PPA.* ***B,*** *The effect of Session (Pre, Post) following OPA stimulation is overlaid onto the same views as A and thresholded on the interaction shown in A. Cold colors represent a decrease in response following TBS of OPA, with hot colors representing an increase. Local decreases are evident inside OPA and posterior PPA.* ***C,*** *Same as B but for OFA stimulation. Here, positive responses are present within OPA. On the ventral surface, responses are negative in posterior PPA and stronger in magnitude than following OPA stimulation.* ***D,*** *Same as B but for sham condition.*

S4: Whole brain main effects

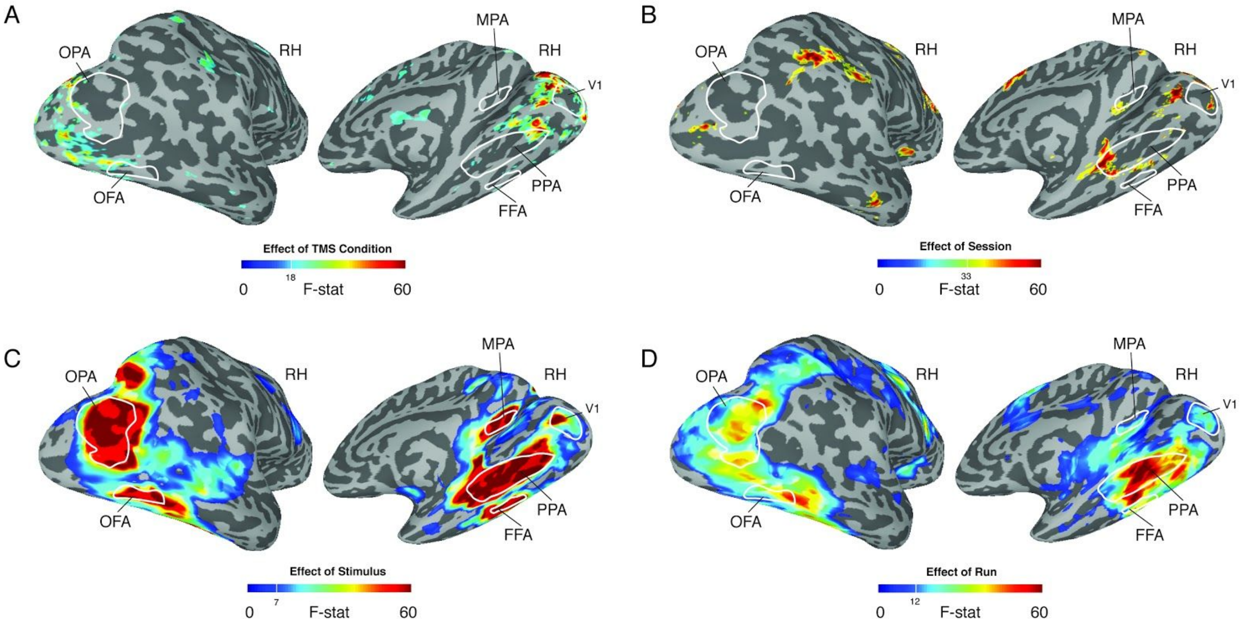

Figure S4: *Main effects at the whole brain level.* ***A,*** *Locations of a significant main effect of TMS condition (p=8.7-9, q=8.2-8) are overlaid onto lateral (left) and medial (right) views of a partially inflated surface reconstruction. Group-based definitions of OPA, OFA, PPA, MPA and FFA are outlined in white. TMS condition effects were largely restricted to EVC, but also overlapped posterior PPA and approached the borders of OPA and OFA.* ***B,*** *Locations of a significant main effect of Session (p=8.7-9, q=8.2-8) are overlaid onto lateral (left) and medial (right) views of a partially inflated surface reconstruction. Group-based definitions of OPA, OFA, PPA, MPA and FFA are outlined in white. Session effects were most prominent in anterior PPA. Additional locations included EVC and to a lesser extent MPA.* ***C,*** *Locations of a significant main effect of Stimulus (p=8.7-9, q=8.2-8) are overlaid onto lateral (left) and medial (right) views of a partially inflated surface reconstruction. Stimulus condition effects were evident throughout visual cortex overlapping all group-based ROI definitions.* ***D,*** *Locations of a significant main effect of Run(p=8.7-9, q=8.2-8) are overlaid onto lateral (left) and medial (right) views of a partially inflated surface reconstruction. Run condition effects were evident throughout visual cortex overlapping all group-based ROI definitions.*

S5: Whole brain linear mixed effects with random slopes

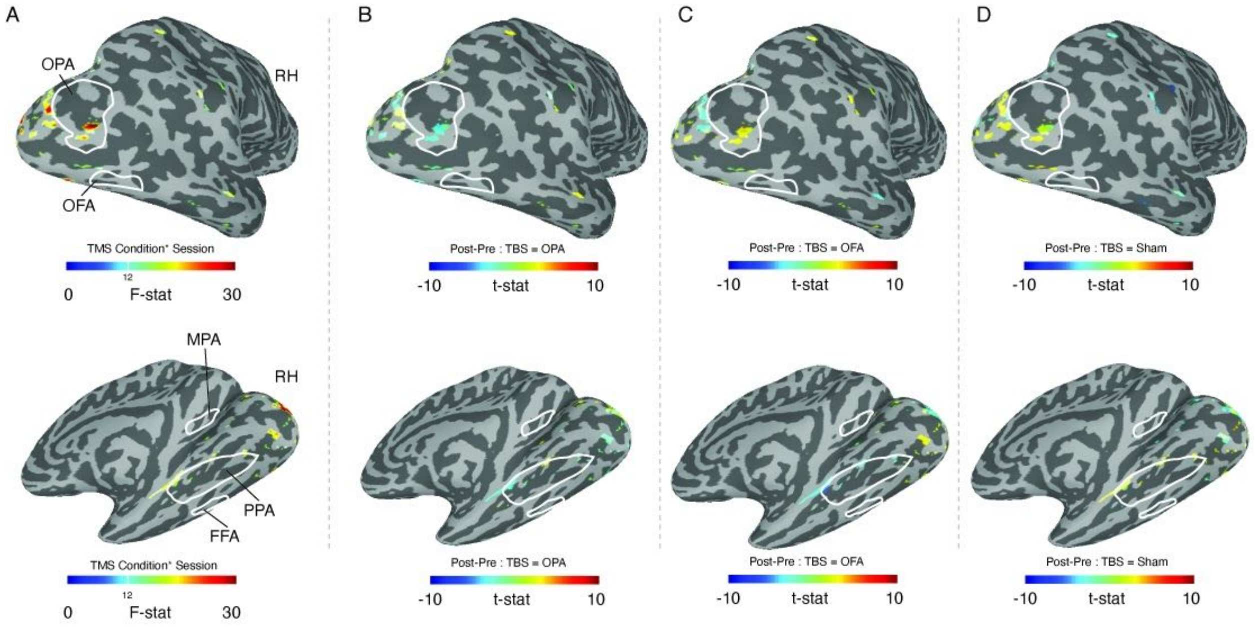

Figure S5: *Linear mixed effects with random slopes.* ***A,*** *Locations of significant Session by TMS Condition interaction effects (p=2.4-6, q=4.2-5) are overlaid onto lateral (top) and medial (bottom) views of a partially inflated surface reconstruction of the right hemisphere (sulci=dark gray, gyri=light gray). Group-based definitions of OPA, OFA (top), PPA, MPA and FFA (bottom) are overlaid in white. Unlike the TMS'd hemisphere, significant interaction effects were both smaller in areal extent and lower in magnitude. Despite this, significant interaction effects were present within EVC, OPA and posterior PPA.* ***B,*** *The effect of Session (Pre, Post) following OPA stimulation is overlaid onto the same views as A and thresholded on the interaction shown in A. Cold colors represent a decrease in response following TBS of OPA, with hot colors representing an increase. Local decreases are evident inside OPA and posterior PPA.* ***C,*** *Same as B but for OFA stimulation. Here, positive responses are present within OPA. On the ventral surface, responses are negative in posterior PPA and stronger in magnitude than following OPA stimulation.* ***D,*** *Same as B but for sham condition.*

S6: Behavioral data (d' and reaction times)

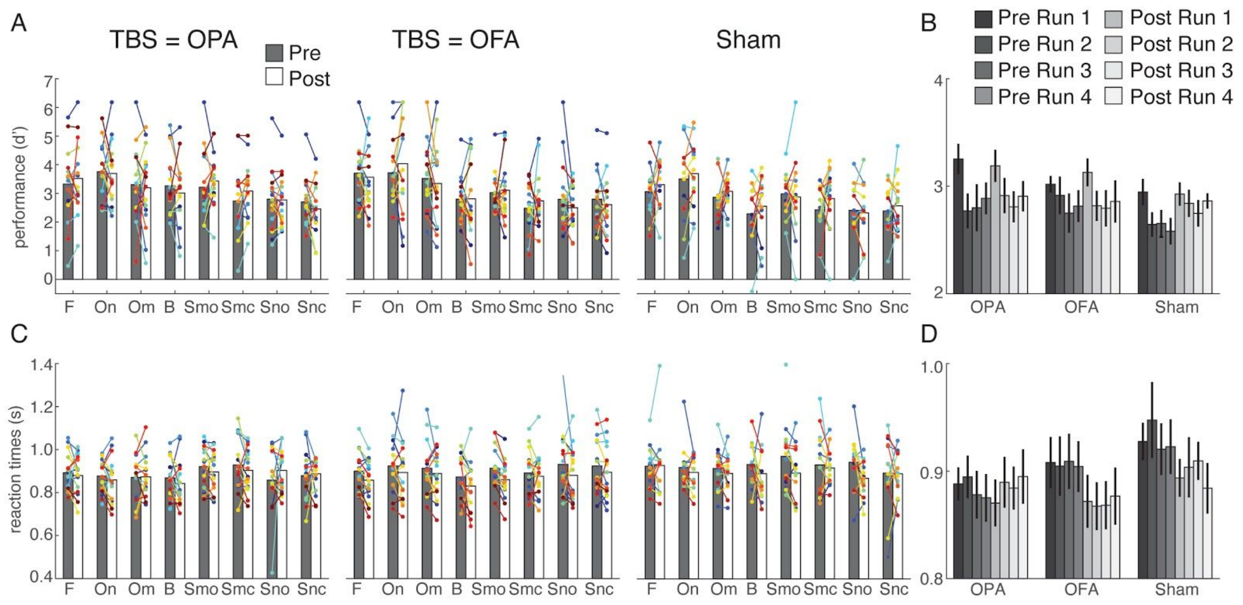

Figure S6: *Behavioral data (d' and reaction times).* ***A****, Bars represent group-average performance ('d) for each stimulus category during Pre (gray) and Post (white) sessions during TBS to OPA (left), TBS to OFA (middle) and the Sham (right). Individual participant data points are overlaid and linked in each case. There was no effect of TMS on d'.* ***B****, Bars represent group-average performance (d') across categories, separated by run. Error bars represent S.E.M. across subjects. There was a main effect of Run on d', indicating decreased performance over time within each Pre and Post session.* ***C-D****, Same as A-B, but showing group-average reaction times (RTs) in seconds. Reaction times were generally lower in the Post compared to the Pre session, but this was not modulated by TMS (no TMS condition by Session interaction).*

S7: Variability in stimulation sites across participants

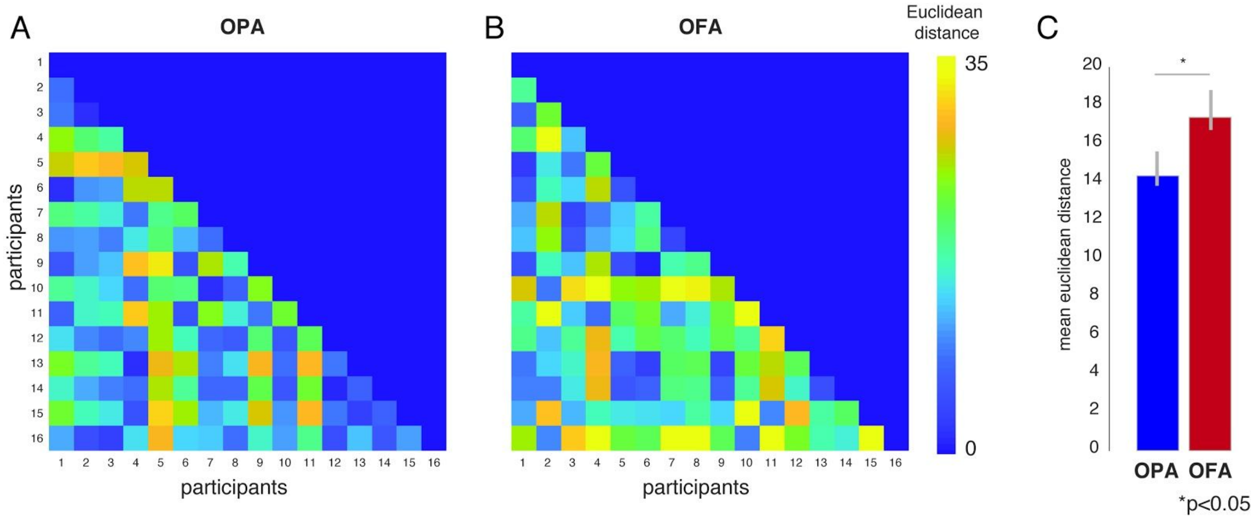

Figure S7: *Variability in stimulation sites across participants.* ***A,*** *Matrix represents the pairwise euclidean distances between rOPA stimulation sites across participants.* ***B,*** *Same as A, but for rOFA.* ***C,*** *Bars represent the mean euclidean distance between participants for OPA (blue) and OFA (red). On average, there was significantly greater variability in target distances across participants for OFA compared to OPA (one-tailed t-test: t(15) = 1.96, p=0.03). Error bars represent standard error of the mean.*

S8: Proximity of OFA and the venous eclipse

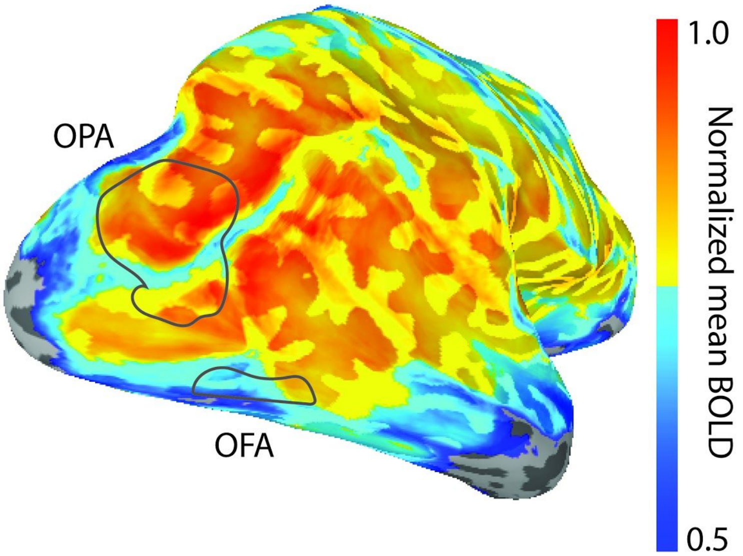

Figure S8: *Proximity of OFA and the venous eclipse. The group-average normalized mean BOLD signal is overlaid onto a partially inflated lateral view of the right hemisphere. Group-based definitions of OPA and OFA are overlaid in gray. The lateral projection of the venous eclipse (area of low-signal) can be seen running along the ventral edge of the brain from posterior-anterior. The ventral boundary of OFA lies in close proximity to the venous eclipse.*

Supplementary References

Brown VA (2020). An approachable introduction to linear mixed effects modeling with implementation in R. *PsyArXiv* https://doi.org/10.31234/osf.io/9vghm

Macmillan, NA & Creelman CD (1991). Detection Theory: A User's Guide (New York: Cambridge University Press).

Meteyard L & Davies RAI (2020). Best practice guidance for linear mixed-effects models in psychological science. *Journal of Memory and Language* 112 104092 https://doi.org/10.1016/j.jml.2020.104092
